# A Patient with Combined CADASIL and MTHFR Homozygosity

**DOI:** 10.1155/2020/4980847

**Published:** 2020-02-17

**Authors:** Sidonie Ibrikji, Tarek El Halabi, Bassem Yamout

**Affiliations:** American University of Beirut Medical Center, Department of Neurology, Beirut, Lebanon

## Abstract

Cerebral Autosomal Dominant Arteriopathy with Subcortical Infarcts and Leukoencephalopathy (CADASIL) is an inherited disorder caused by a mutation in the NOTCH 3 gene, characterized by early onset of subcortical lacunar infarcts in the absence of vascular risk factors and cerebral microbleeds. Homozygosity for the factor Methylenetetrahydrofolate Reductase (MTHFR) is also associated with lacunar stroke risk and cerebral small-vessel disease regardless of the homocysteine level. The coexistence of MTHFR C677T homozygosity and NOTCH 3 mutation has never been reported in the literature previously, and that brings up the challenge of antithrombotic treatment in the presence of cerebral microbleeds.

## 1. Introduction

This is the case of a 49-year-old man presenting for stepwise speech impairment and progressive mental slowing of 14 years duration. In his early 30s, he developed recurrent attacks of migraine, preceded by visual aura, for which he was symptomatically treated. At the age of 35, he had a left posterior frontal subcortical ischemic infarct resulting in heavy speech that progressively improved with time. At the age of 37, another subcortical ischemic infarct occurred in his right centrum semioval, leading to left-sided hemiparesis with partial recovery. Three years prior to his presentation, a second left frontotemporal stroke led to severe dysarthria with only minimal recovery. All through this period, he reported mild forgetfulness and mental slowing in retrieving ideas. He had no vascular risk factors except for heavy smoking (more than a 100-pack-year). His family history was significant for a 30-year-old daughter with migraines without aura, and a 25-year-old son with unclear seizure disorder.

On neurological exam, he had moderate flaccid dysarthria and left-sided hemiparesis. His MMSE (Mini-Mental Status Exam) score was 26/30; showing intact attention, orientation, and registration, while losing points on memory (2 out 3 objects recalled at 5 minutes), visuospatial capacities, repetition, and writing.

**Figure 1:**
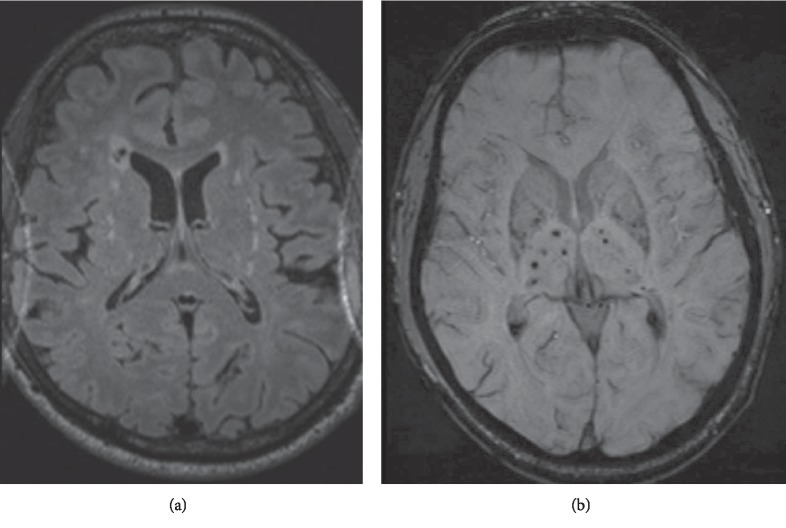
MRI brain showing abnormal FLAIR signal (a) along the bilateral external capsules, with abnormal hypo intensities in bilateral basal ganglia on T2 FFE (b).

Cerebrospinal fluid studies (CSF) taken from a lumbar puncture were unremarkable. Magnetic Resonance Imaging (MRI) of the brain showed high T2W lesions involving the external capsules and anterior temporal poles bilaterally, multiple old subcortical infarcts involving predominately the frontal lobes, and bilateral low signal lesions on Susceptibility Weighted Imaging (SWI), in the basal ganglia and thalami suggesting microhemorrhages (Figure 1). Magnetic Resonance Angiography (MRA) of the cervical and cerebral vessels was normal.

A hypercoagulable panel and Cerebral Autosomal Dominant Arteriopathy with Subcortical Infarcts and Leukoencephalopathy (CADASIL) genetic testing were ordered and revealed a homozygous Methylenetetrahydrofolate Reductase (MTHFR) C677T mutation with a normal homocysteine level (10 *µ*mol/L) and a NOTCH3 gene mutation (Heterozygous for c.3691CT p.Arg1231Cys). The patient was on folate, vitamin B12, and vitamin B6 supplements, with normal serum folate and vitamin B12 levels.

Given the presence of microbleeds on MRI and the high risk they confer for bleeding, the patient was not put on antithrombotic treatment. A follow-up 6 months later revealed no new strokes, and the patient was physically and cognitively unchanged.

## 2. Discussion

CADASIL is a rare, adult-onset inherited disorder, with a mean age at onset of 36.7 years. The earliest clinical manifestation is migraine with aura, followed by subcortical lacunar ischemic events, considered as the most common manifestation, occurring in 60 to 85% of cases, in the absence of conventional vascular risk factors [1]. Cognitive decline is the second most frequent symptom, and consists of impairment in attention and memory, alteration in executive function, and later on affecting language, reasoning, and verbal and visual memory. Apathy is less common, occurring in 40% of cases. Abnormalities on brain MRI are present in virtually all individuals carrying the mutation after the age of 35 years and consist of high-FLAIR signals indicating leukoencephalopathy, predominating in the periventricular areas, centrum semioval, anterior temporal poles, and external capsules. The 2 latter MRI abnormalities have the highest diagnostic sensitivity, nearing 90%. Cerebral microbleeds are also commonly seen in cortical and subcortical areas, white matter, brainstem, and thalamus [2]. Treatment focuses on primary and secondary prevention by controlling vascular risk factors and smoking avoidance. The benefit of aspirin alone or in combination with clopidogrel in secondary stroke prevention in CADASIL is still questionable. In the presence of cerebral microbleeds, the risk of aspirin-associated intracranial bleed increases, but is still much lower than the rate of ischemic attacks [3].

Pathophysiologically, CADASIL is caused by a mutation in the NOTCH3 gene on chromosome 19. NOTCH3 contributes to arterial development through a cell-surface protein expressed on vascular smooth muscle cells, playing a major role in the stability of those cells' phenotype [4]. The patient can be homozygous or heterozygous for the mutation, homozygosity predicting a slightly more severe course whether clinically or radiologically [5].

Furthermore, MTHFR polymorphism increases the risk of lacunar stroke risk and cerebral small-vessel disease, especially in association with hypertension, but can occur in the absence of other vascular risk factors [6]. The MTHFR mutation reduces the activity of the enzyme responsible for the remethylation of homocysteine to methionine, elevating the plasma concentrations of homocysteine, an independent risk factor for small-vessel ischemic disease through endothelial dysfunction [4]. Our patient had normal homocysteine levels, probably secondary to supplementation with folic acid, vitamin B12, and vitamin B6. Lowering homocysteine levels did not demonstrate a significant effect in decreasing the risk of stroke [7].

The coexistence of MTHFR C677T homozygosity and NOTCH 3 mutation has never been reported in the literature previously and increases the risk of small-vessel ischemic strokes and silent brain lesions, secondary to a combination of endothelial dysfunction on top of microangiopathic changes.
